# pH electrode performance under automated management conditions

**DOI:** 10.1155/S1463924697000266

**Published:** 1997

**Authors:** J. E. A. Comer, C. Hibbert

**Affiliations:** Sirius Analytical Instruments Ltd Riverside Forest Row Business Park Forest Row East Sussex RH18 5DW UK

## Abstract

pH is frequently measured in laboratories, but to have confidence in the results it is necessary to know that it was measured properly. For an electrode to give accurate results it must be treated well and calibrated correctly. In this paper, an automated system for pH measurement is described; the system uses the operational pH scale and calibrates using two or three buffer solutions, taking proper account of the effects of temperature on the system. The system can be programmed with standard methods and procedures to ensure that the electrode gives the best possible performance. Calibrations and measurements within the system are reproducible, and the automated system is more robust than the manual pH meter, and requires less operator time.


Journal of Automatic Chemistry, Vol. 19, No. 6 (November-December 1997) pp. 213-224

### pH electrode performance under automated management conditions

**J. E. A. Comer and C. Hibbert***

*Sirius Analytical Instruments Ltd, Riverside, Forest Row Business Park, Forest Row, East Sussex RH18 5DW, UK*

*pH is frequently measured in laboratories, but to have confidence in the results it is necessary to know that it was measured properly. For an electrode to give accurate results it must be treated well and calibrated correctly. In this paper, an automated system for pH measurement is described; the system uses the operational pH scale and calibrates using two or three buffer solutions, taking proper account of the effects of temperature on the system. The system can be programmed with standard methods and procedures to ensure that the electrode gives the best possible performance. Calibrations and measurements within the system are reproducible, and the automated system is more robust than the manual pH meter, and requires less operator time.*

### Introduction pH is one of the most commonly measured chemical parameters. It is usually measured with a glass electrode and reference half-cell connected to a pH meter, which converts the mV output from the electrodes to a pH value. The principles of pH measurement are well established [1, 2], and the construction and mechanisms of pH electrodes is also well understood [3]. Unfortunately, there is a tendency for pH measurement to be regarded as 'trivial', and the work is often entrusted to non-analysts or poorly trained personnel [4]. This is not satisfactory, since the correct measurement involves many steps which must be carried out methodically and in sequence to achieve an accurate and reproducible pH measurement [5]. These steps include topping up the electrode filling solution, changing the buffer solutions, calibrating the electrode regularly, cleaning the electrode properly between solutions, taking temperature effects into account [6], and applying personal judgement as to the attainment of a stable reading. Inattention to one or more of these steps leads to errors in measurement, which often shows up as inter-operator or inter-laboratory inconsistencies.

In view of the importance of pH measurement in industrial quality control applications, the authors developed an automated system for pH measurement that incorporates a temperature probe and stirrer alongside the electrode in a specially designed electrode holder. This automated approach reduces the errors associated with manual measurement, and provides traceability for good laboratory practice (GLP). Unlike other systems, which have been based on commercially available laboratory robots [7, 8], this is an integrated system, dedicated to pH measurement.

The system uses the operational pH scale and the conventional method of pH calibration with two or three buffer solutions. Two vials of each bufffer are used. The first vial is used for washing the electrode, and the second is used for calibrating. This procedure minimizes carry-over of wash water into the calibration buffers [9]. The readings are taken automatically after a user-set time or when user-programmed stability criteria have been reached. The instrument has a built-in table of buffer pH vs temperature data. The electrode is washed in two wash beakers containing deionized water before reading the pH samples or buffers. When it is not in use, the electrode is stored in pH 7 buffer. Figure 1 shows the positions of the buffer vials and wash beakers within the system.

The system can be programmed with reminders to prompt the user to change the buffers and wash waters at a fixed time each day, and to top up the electrode filling solution at regular intervals. Standard methods and procedures can be programmed to ensure that measurements are always carried out under exactly the same conditions, and calibrations are performed at time intervals with a choice of recognized buffers. Sample measurements and calibrations are logged and deviations from the programmed methods are recorded. User names can also be programmed and personal identification numbers (PIN) can be used to prevent access by unauthorized personnel. Calibration and sample data can be accessed via an audit menu, and displayed in graphical or tabular form. An audit trail log is automatically printed when reminders and actions are acknowledged, and when methods are created or amended. Sample results are also printed. If a reminder to install fresh pH buffers or wash solutions appears when the sytem is programmed to calibrate automatically, then the system will not calibrate until the reminder is acknowledged and the action has been performed. Out-of-range parameters can be set for electrodes, buffers and calibrations, and a warning message appears if the electrode or buffers deviate from acceptable behaviour.

In this study the long-term performance of the system was investigated by making a series of automated calibrations using different electrodes, and a series of measurements of pH of typical samples.

### Experimental

### *Equipment and materials*

The automated pH system used was the single-sample version of the Sirius GLpH. The manual pH meters used for the comparison study were the 720A and 920A

* Author to whom correspondence should be addressed.

0142-0453/97 $12·00 © 1997 Taylor & Francis Ltd

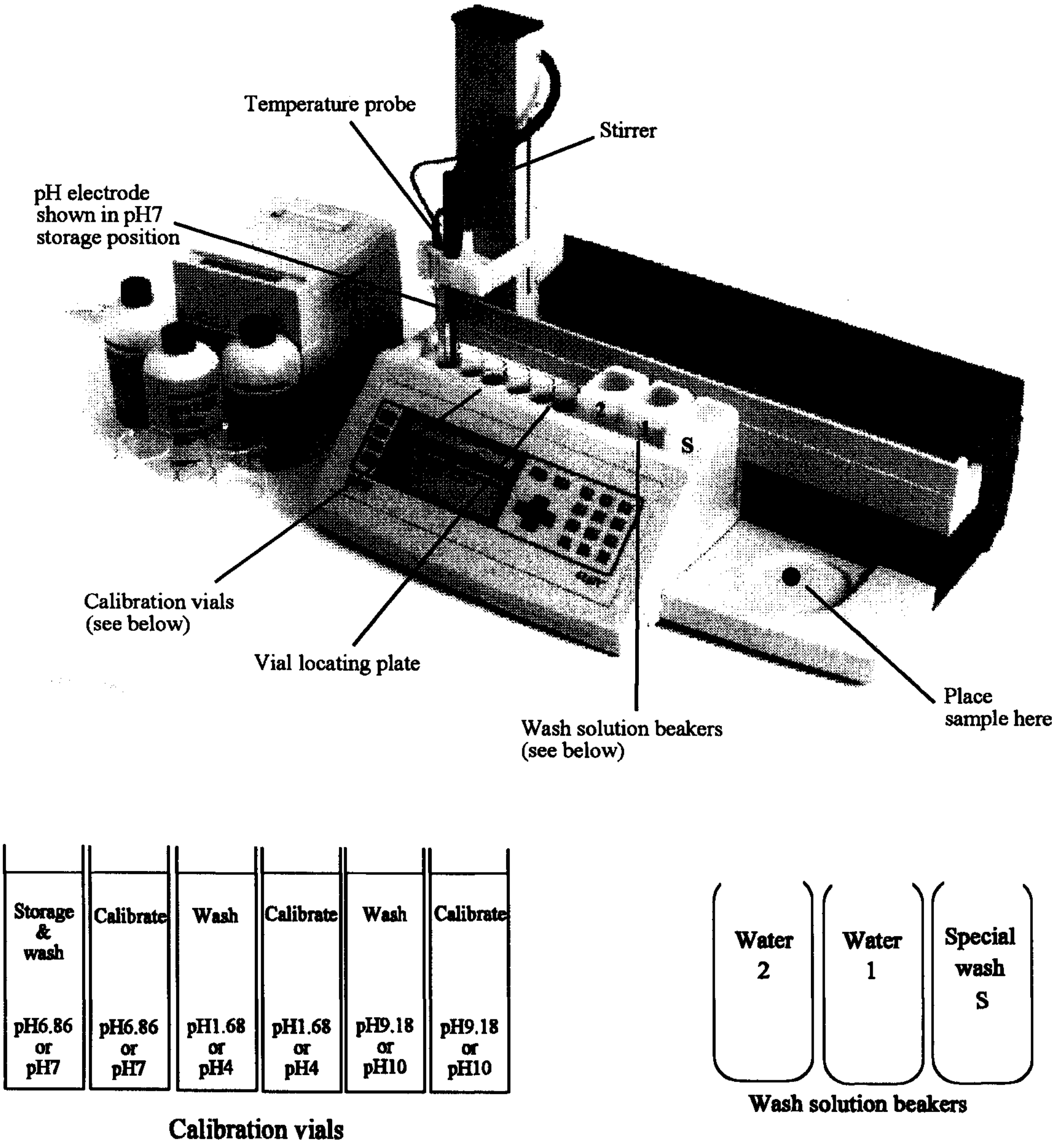

*Figure 1. Position of buffer vials and wash beakers in the automated pH measurement system.*

models from ORION Research Inc. (Beverley, MA, USA). A cantilever stand and glass bodied ATC probe (part number 917006) from ORION were used with the manual pH meters. The Ag/AgCl ceramic junction and Ag/AgCl sleeve junction electrodes were made by Sentek (Braintree, UK). ORION pH 4·01, 7·00 and 10·01 buffers traceable to NIST standard reference materials were used. The mouthwash, tomato ketchup and shampoo were packaged products from well-known manufacturers. The paint sample was kindly donated by a well-known manufacturer of automotive paint. The non-ionic detergent used was Triton X-100 which is a registered trademark of Rohm & Haas.

*Methodology*

Using a pH electrode under automated conditions leads to improved accuracy and precision, because of the reproducible way in which the measurements are made. A study of the calibration data produced under these conditions was carried out over a period of two months. The automated pH measurement system was fitted with a Ag/AgCl double junction pH electrode with a ceramic junction, and pH 4, 7 and 10 buffers were installed. The two wash beakers were filled with fresh deionized water every morning. The special wash beaker was not used during the calibration procedure. The buffers were also changed every morning, and the system was programmed to calibrate in the three buffers every hour. The ceramic junction electrode was replaced with a Ag/AgCl double junction pH electrode with a sleeve junction after 16 days due to a deterioration in calibration performance, which was discovered during this study (figure 2).

Each hourly automated calibration session was done according to a standardized procedure as follows: the electrode was moved from its storage position in the first (left hand) vial of the pH 7 buffer to the second (right

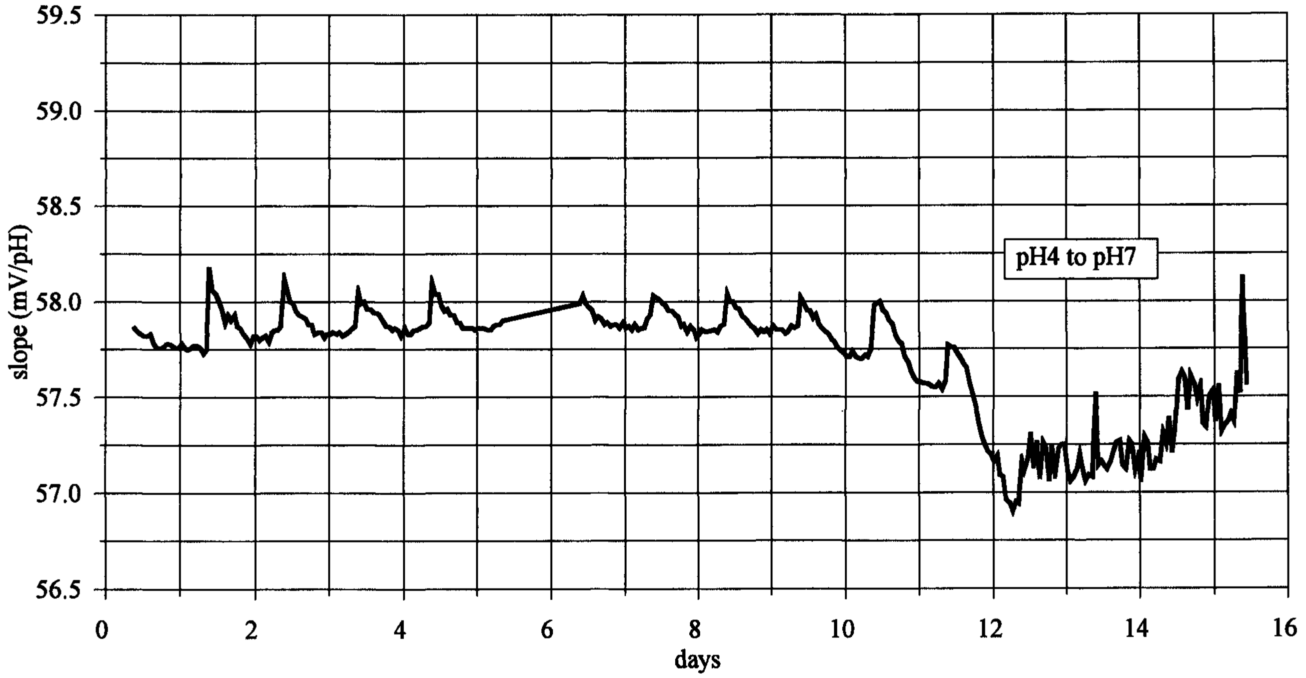

*Figure 2. Slope between pH4 and pH7 buffers for the Ag/AgCl ceramic junction electrode showing deterioration after day 11.*

hand) vial of the pH 7 buffer. The buffer solution was stirred for 5 s, after which stirring was turned off and the reading taken after stability had been reached. The electrode was then rinsed in each of the two wash beakers for 5 s to remove carry-over of buffer. The electrode was then moved to the first vial of pH 4 buffer for washing. The buffer was stirred for 5 s then the electrode was moved to the second vial of pH 4 buffer for calibrating. The buffer solution was stirred for 5 s, after which the stirring was turned off and the reading taken after stability had been reached. The electrode was again rinsed in each of the two wash beakers for 5 s before it moved to the first vial of pH 10 buffer for washing. The buffer was stirred for 5 s then the electrode was moved to the second vial of pH 10 buffer for calibrating. The buffer solution was stirred for 5 s, after which the stirring was turned off and the reading taken after stability was reached. The electrode was then rinsed again in each wash beaker before being returned to the storage position.

Two samples (mouthwash and shampoo) were measured with the Ag/AgCl ceramic junction electrode. Four samples (mouthwash, shampoo, tomato ketchup, and a water-based automotive paint) were measured with the Ag/AgCl sleeve junction electrode. The measurements were made in triplicate at hourly intervals. For comparison, these four samples were also measured using a manual pH meter.

The antiseptic mouthwash had a thin consistency and tended to foam when stirred vigorously. Therefore a slow stirrer speed was selected. The sample was stirred for 2 s, after which stirring was turned off and the reading taken after stability had been reached. After measurement the electrode waited above the sample for 2 s to allow drips to fall and was then washed in each wash beaker for 5 s. The special wash beaker was not used. A fresh mouthwash sample was taken each morning and it was kept covered during the day to avoid evaporation.

When a viscous sample consistency is chosen in the method, the stirrer is automatically set to a slow speed. This is the case with the anti-dandruff shampoo sample. The electrode was slowly moved up and down twice when it first entered the sample to ensure that it was properly covered by the sample. The sample was then stirred for 5 s, after which stirring was turned off and the reading taken after stability was reached. After each measurement the electrode waited above the sample for 35 s (the drip drop time) to allow drips to fall before being washed in the special wash beaker containing 5% of non-ionic detergent for 15 s. It was then washed in the two wash water beakers for 15 s.

Tomato ketchup was also a viscous liquid, so a similar method to shampoo was used. However, the sample drip drop time was reduced to 5 s as ketchup tends not to drip. After each measurement the electrode was washed in the special wash beaker containing 5% of non-ionic detergent for 25 s, then in the two wash water beakers for 10 s.

The water-based automotive paint had a thin consistency, but contained fine particulate material. A normal stirrer speed was selected, but this caused the paint to froth, so the method was modified to use a slow speed. The sample was stirred for 5 s, after which stirring was turned off and the reading taken after stability was reached. The sample drip drop time was set at 5 s. After each measurement the electrode was washed in the special wash containing 2-butoxyethanol for 30 s, then in the two wash water beakers for 15 s.

For comparison, measurements were also carried out using a manual pH meter with a Ag/AgCl sleeve junction electrode, automatic temperature compensation (ATC) probe, stand and magnetic stirrer. Calibrations were performed with pH 4, 7 and 10 buffers. Small beakers were chosen to minimize the volumes of buffer solutions being used. The electrode was rinsed with deionized water, then with the relevant buffer before calibration in each buffer. The buffer solution was stirred for 5 s, after which stirring was turned off and the reading taken after stability was reached. Fresh buffers were used each day.

The four samples were measured using methods similar to those used on the automated pH system. The measurements were made directly after a calibration had been performed. The ketchup and shampoo samples were not stirred, but the electrode was slowly moved up and down in the sample to ensure that the electrode junction was covered properly. After measurement, the electrode was washed in a beaker containing 5% of non-ionic detergent until clean then rinsed with deionized water. The paint sample was stirred slowly to avoid frothing, however, sediment could be seen settling on the bottom of the beaker. The electrode was washed in 2-butoxyethanol until clean, then rinsed with deionized water.

### Results and discussion

### *Electrode stability*

Figures 2 and 3 show the calibration data for the Ag/AgCl ceramic junction electrode over the 16-day period of the study. This electrode had been in use for several weeks before the start of this study. The calibration slopes gradually drift downwards over a period of one day and sharply increase when fresh buffers are installed each morning. This drift is more marked between the pH 7 and pH 10 buffers, presumably because pH 10 solutions absorb carbon dioxide from the air and start to degrade after several hours of use. These graphs show how important it is to change the buffers regularly. The sharp change in the slope between five and seven days was due to the fact that the buffers were not changed on the Saturday morning when prompted by the reminder, and as a result no calibrations were performed until the buffers were changed on the Sunday morning.

The calibration data became less reproducible after approximately 10 days, particularly between the pH 4 and pH 7 buffers. This corresponds with the start of pH measurements in a viscous sample of shampoo. A possible reason for this is that the electrode junction may have become clogged up with sample. Figure 4 shows the hourly millivolt (mV) and temperature readings in the pH 7 buffer for the Ag/AgCl ceramic junction electrode. The deterioration in the electrode performance can clearly be seen here, and gives a better indication of the electrode performance than the slopes. In an attempt to restore its performance, the ceramic junction electrode was cleaned out and refilled with filling solution, but no improvement was observed. The electrode was removed and a sleeve junction electrode installed.

Figures 5 and 6 show the calibration data for the Ag/AgCl sleeve junction electrode. A similar downward drift throughout the day is observed and, again, this is more marked between the pH 7 and pH 10 buffers. The buffers were not changed for the last four days of the study. Figure 6 indicates that the electrode will very quickly deviate from acceptable behaviour if the buffers are not changed for a few days. Figure 7 reveals that the slopes are constant if buffers are changed every day, but the pH 7 to pH 10 slope decreases if the buffers are not changed.

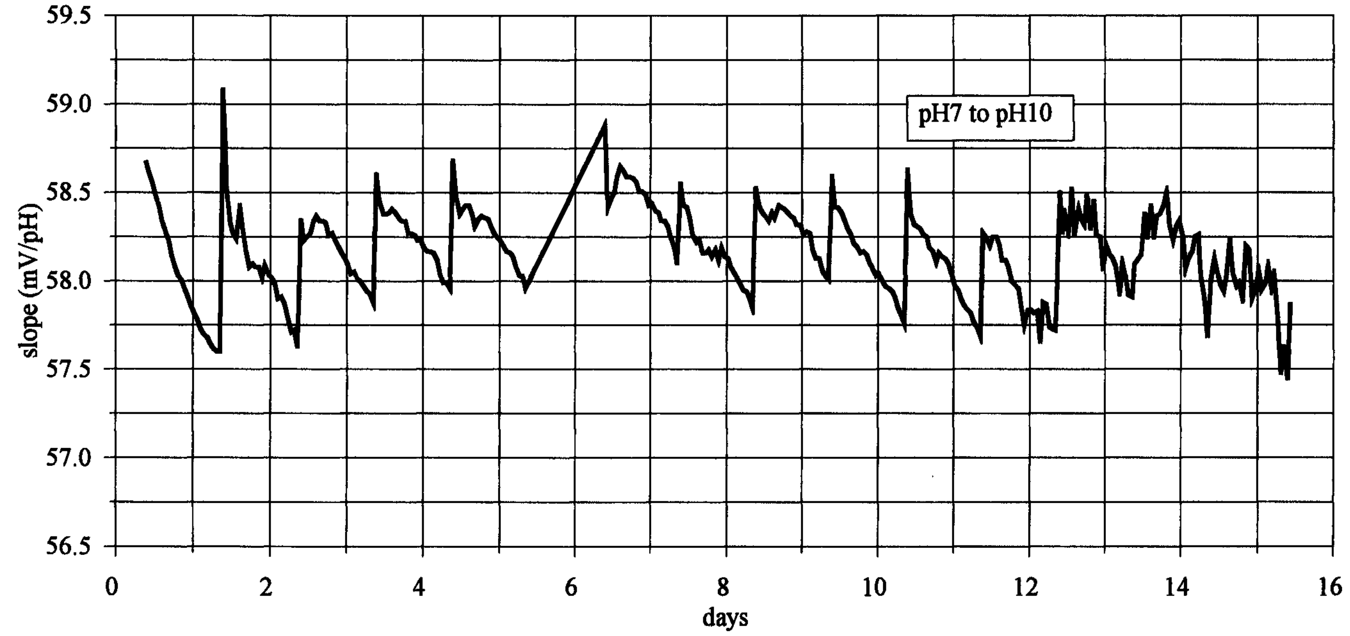

*Figure 3. Slope between pH 7 and pH 10 buffers for the Ag/AgCl ceramic junction electrode showing deterioration after day 11.*

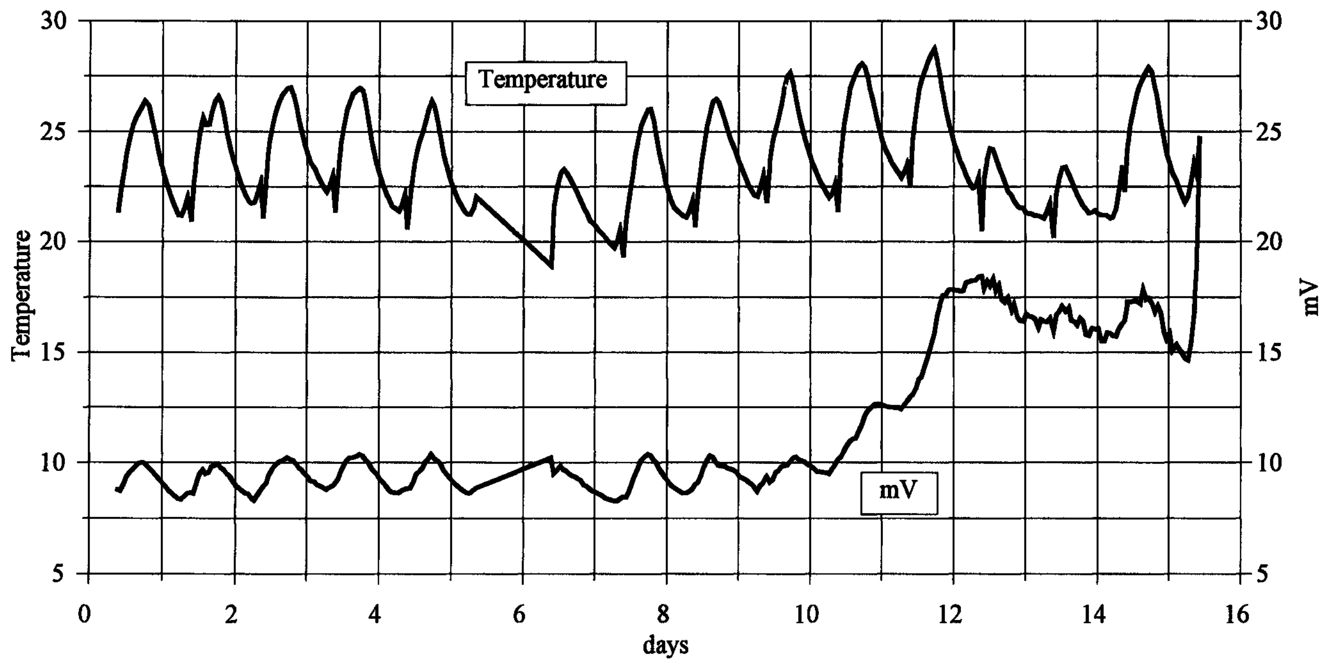

*Figure 4. Millivolts and temperature in pH 7 buffer for the Ag/AgCl ceramic junction electrode showing deterioration after day 11.*

*Temperature effects*

In all mV graphs a dependence on temperature can also be observed. Figure 4 shows the hourly mV and temperature readings in the pH 7 buffer for the Ag/AgCl ceramic junction electrode. The temperature gradually increases and reaches a peak at *ca.* 5.30 p.m. or 6.30 p.m. before slowly decreasing and reaching a low point 12 h later. The temperature starts to increase again for a few hours before fresh, cool buffers are installed at approximately 9.30 a.m. causing the temperature to drop sharply before increasing again. The peak temperature is significantly lower at weekends than during the week. The peaks and troughs of the mV graph match those from the temperature graph to within an hour. The temperature changes are caused by the laboratory heating system, which is turned off at night and weekends. Although temperature clearly affects the raw mV data, the use of ATC minimizes its effect on measured pH values, and a temperature-controlled environment is not necessary, provided temperature is reported alongside each pH measurement.

Figure 8 shows the hourly mV and temperature readings in the pH 7 buffer for the Ag/AgCl sleeve junction electrode. The temperature peaks at *ca.* 5–6 p.m. and reaches its lowest point at approximately 6–7 a.m. The same drop in temperature previously noted is observed when the buffers are changed each morning. Although the mV reading again indicates a dependence on temperature, the readings remain stable in the long term. It can be seen by the deviations from the average mV reading how the electrode was affected by viscous and more difficult samples. The electrode, however, always recovered quickly, usually by the next calibration an hour later. The large deviation on the 28th day arose because the instrument was turned off overnight after a demonstration, while the electrode was left in one of the wash beakers.

*pH measurements*

The results for the four samples taken using the automated pH system are presented in tables 1–4. A total of 69 measurements were taken of the mouthwash sample with the Ag/AgCl ceramic junction electrode. The mean triplicate pH values range from 6·913 to 6·942. The shampoo sample was also measured with the Ag/AgCl ceramic junction electrode and 63 measurements were made. The mean pH values range from 6·226 to 6·310. The tomato ketchup and water-based paint samples were measured with the Ag/AgCl sleeve junction electrode. Some 48 measurements were made in the ketchup sample and the mean pH values range from 3·471 to 3·506. A total of nine measurements were made in the paint sample and the mean triplicate pH values range from 6·136 to 6·192.

Tables 5 and 6 show the calibration data for the automated pH system and manual laboratory pH meter. The automated pH system provides two slopes for each calibration, while the manual meter only gives one. Table 7 compares the volumes of buffers used throughout the study. Although small beakers were selected for use with the manual pH meter, it was still necessary to use 50 ml of each buffer to ensure that the electrode junction was properly covered. While 40 ml of each buffer is needed initially to set up the automated pH system, only the 20 ml of each buffer used for the calibration need to be changed each day, as the previous day's calibration buffers can then be used for the pre-calibration wash procedure. On the second day of the study, one of the beakers being used with the manual pH meter was accidentally knocked over and had to be refilled with fresh buffer. This is unlikely to happen with the automated pH system, because the buffer vials and wash beakers are held firmly in place within the instrument.

Table 8 compares the length of time taken to perform

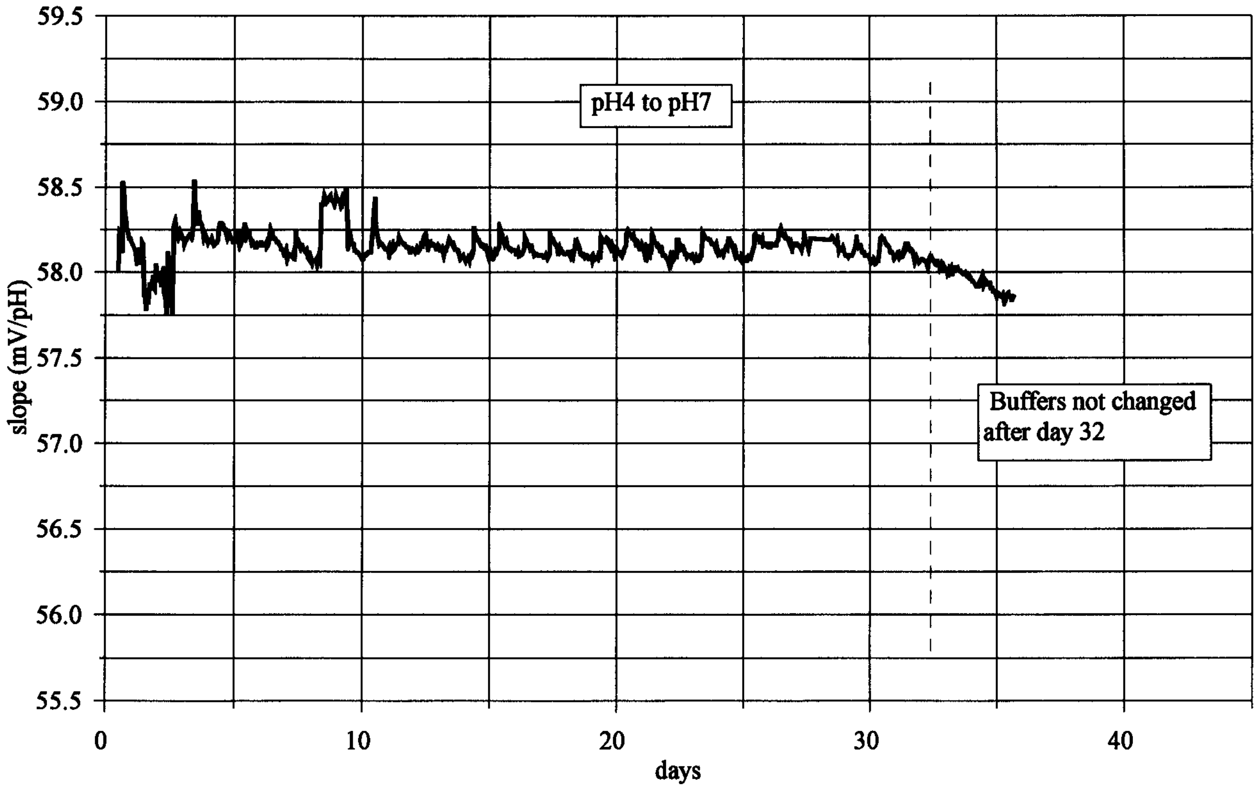

*Figure 5. Slope between pH4 and pH7 buffers for the Ag|AgCl sleeve junction electrode.*

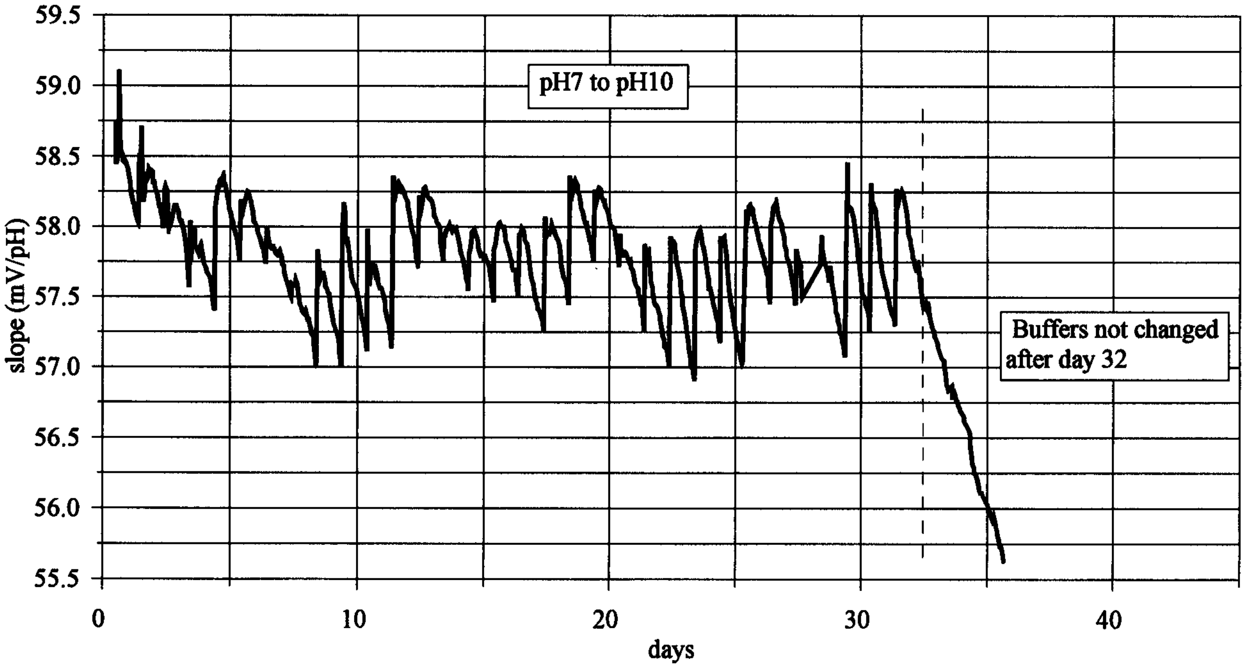

*Figure 6. Slope between pH7 and pH10 buffers for the Ag|AgCl sleeve junction electrode.*

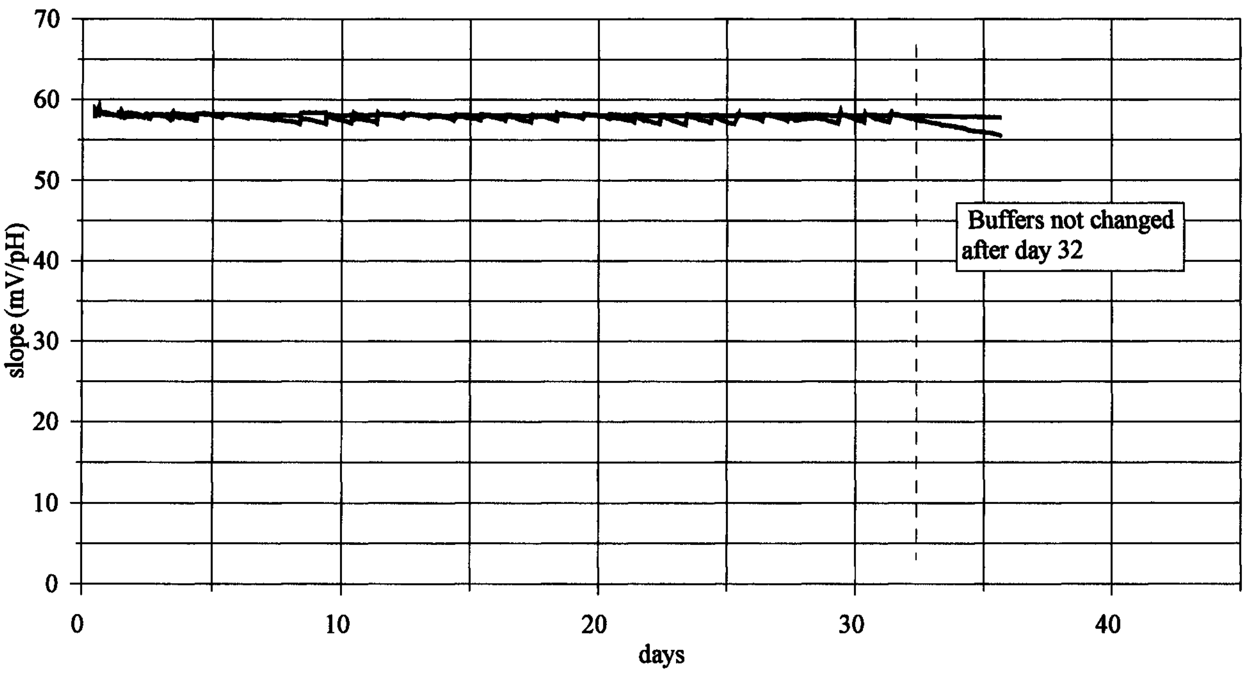

*Figure 7. Calibration slopes for Ag|AgCl sleeve junction electrode.*

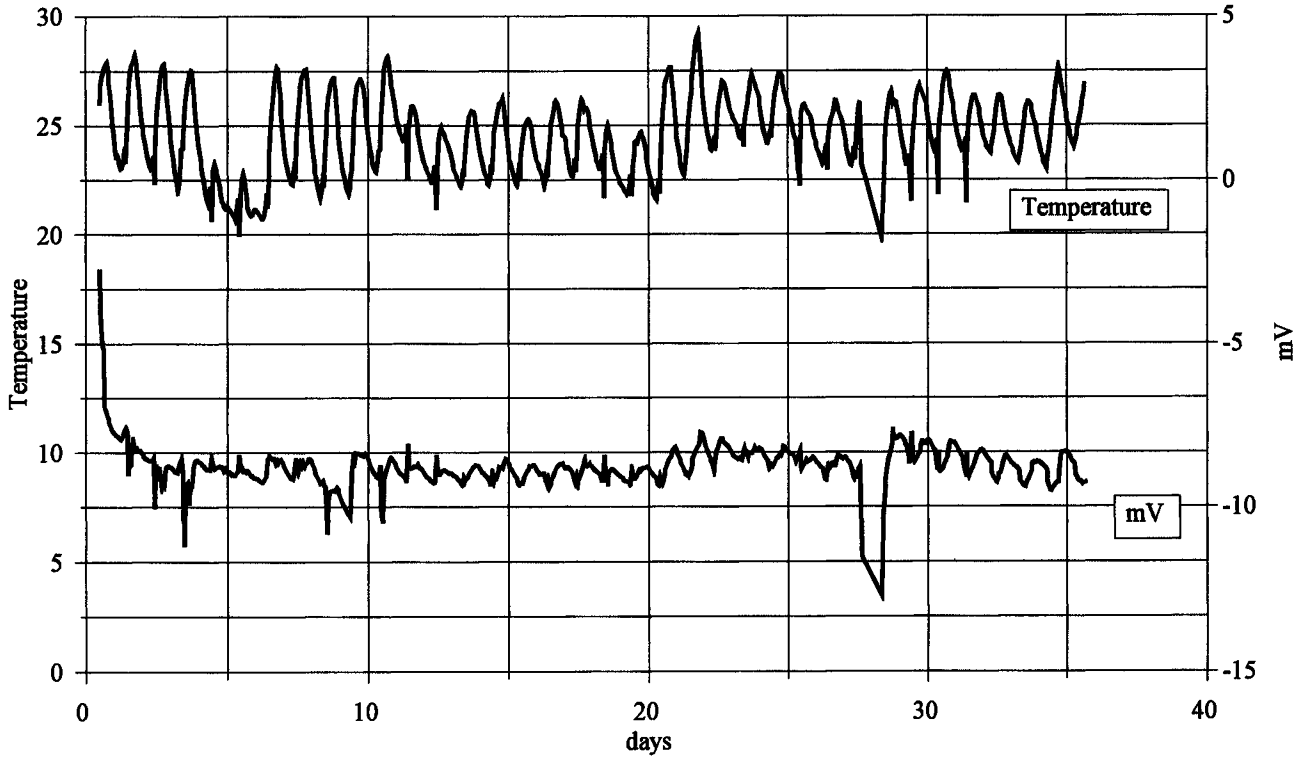

*Figure 8. Millivolts and temperature in the pH7 buffer for the Ag|AgCl sleeve junction electrode.*

*Table 1. pH measurements of mouthwash with a Ag|AgCl ceramic junction electrode.*

| Date | pH 1 | pH 2 | pH 3 | Average temp (°C) | Mean | SD |
|---|---|---|---|---|---|---|
| 4/2/97 | 6·931 | 6·927 | 6·925 | 22·5 | 6·928 | 0·003 |
| 4/2/97 | 6·933 | 6·928 | 6·927 | 22·8 | 6·929 | 0·003 |
| 4/2/97 | 6·929 | 6·926 | 6·924 | 22·6 | 6·926 | 0·003 |
| 5/2/97 | 6·924 | 6·923 | 6·919 | 22·0 | 6·922 | 0·003 |
| 5/2/97 | 6·928 | 6·924 | 6·924 | 22·7 | 6·925 | 0·002 |
| 5/2/97 | 6·926 | 6·918 | 6·922 | 23·3 | 6·922 | 0·004 |
| 5/2/97 | 6·921 | 6·917 | 6·915 | 23·6 | 6·918 | 0·003 |
| 6/2/97 | 6·921 | 6·909 | 6·910 | 19·9 | 6·913 | 0·007 |
| 6/2/97 | 6·923 | 6·911 | 6·913 | 21·2 | 6·916 | 0·006 |
| 6/2/97 | 6·923 | 6·916 | 6·916 | 22·3 | 6·918 | 0·004 |
| 6/2/97 | 6·922 | 6·913 | 6·916 | 23·0 | 6·917 | 0·005 |
| 6/2/97 | 6·926 | 6·914 | 6·914 | 23·4 | 6·918 | 0·007 |
| 6/2/97 | 6·924 | 6·913 | 6·903 | 23·5 | 6·913 | 0·011 |
| 6/2/97 | 6·922 | 6·917 | 6·912 | 24·0 | 6·917 | 0·005 |
| 6/2/97 | 6·924 | 6·919 | 6·918 | 24·4 | 6·920 | 0·003 |
| 7/2/97 | 6·935 | 6·928 | 6·922 | 21·1 | 6·928 | 0·007 |
| 7/2/97 | 6·937 | 6·931 | 6·931 | 22·1 | 6·933 | 0·003 |
| 7/2/97 | 6·940 | 6·932 | 6·928 | 23·0 | 6·933 | 0·006 |
| 7/2/97 | 6·942 | 6·935 | 6·936 | 23·5 | 6·938 | 0·004 |
| 7/2/97 | 6·945 | 6·938 | 6·934 | 23·8 | 6·939 | 0·006 |
| 7/2/97 | 6·947 | 6·925 | 6·937 | 23·9 | 6·936 | 0·011 |
| 7/2/97 | 6·947 | 6·942 | 6·936 | 24·5 | 6·942 | 0·006 |
| 7/2/97 | 6·950 | 6·936 | 6·937 | 24·7 | 6·941 | 0·008 |

*Table 2. pH measurements of shampoo with a Ag|AgCl ceramic junction electrode.*

| Date | pH 1 | pH 2 | pH 3 | Average temp (°C) | Mean | SD |
|---|---|---|---|---|---|---|
| 5/2/97 | 6·229 | 6·230 | 6·224 | 21·6 | 6·228 | 0·003 |
| 5/2/97 | 6·226 | 6·238 | 6·244 | 22·2 | 6·236 | 0·009 |
| 5/2/97 | 6·234 | 6·229 | 6·235 | 22·8 | 6·233 | 0·003 |
| 5/2/97 | 6·227 | 6·232 | 6·233 | 23·3 | 6·231 | 0·003 |
| 5/2/97 | 6·228 | 6·224 | 6·227 | 23·4 | 6·226 | 0·002 |
| 6/2/97 | 6·239 | 6·239 | 6·251 | 20·3 | 6·243 | 0·007 |
| 6/2/97 | 6·244 | 6·240 | 6·242 | 21·4 | 6·242 | 0·002 |
| 6/2/97 | 6·246 | 6·247 | 6·256 | 22·3 | 6·250 | 0·006 |
| 6/2/97 | 6·254 | 6·254 | 6·255 | 22·9 | 6·254 | 0·001 |
| 6/2/97 | 6·254 | 6·253 | 6·256 | 23·3 | 6·254 | 0·002 |
| 6/2/97 | 6·254 | 6·257 | 6·254 | 23·7 | 6·255 | 0·002 |
| 6/2/97 | 6·254 | 6·258 | 6·256 | 24·0 | 6·256 | 0·002 |
| 6/2/97 | 6·253 | 6·254 | 6·262 | 24·2 | 6·256 | 0·005 |
| 7/2/97 | 6·277 | 6·273 | 6·278 | 21·5 | 6·276 | 0·003 |
| 7/2/97 | 6·284 | 6·280 | 6·282 | 22·2 | 6·282 | 0·002 |
| 7/2/97 | 6·282 | 6·279 | 6·282 | 23·0 | 6·281 | 0·002 |
| 7/2/97 | 6·283 | 6·285 | 6·292 | 23·4 | 6·287 | 0·005 |
| 7/2/97 | 6·289 | 6·291 | 6·293 | 23·5 | 6·291 | 0·002 |
| 7/2/97 | 6·301 | 6·299 | 6·296 | 24·0 | 6·299 | 0·003 |
| 7/2/97 | 6·303 | 6·301 | 6·301 | 24·5 | 6·302 | 0·001 |
| 7/2/97 | 6·312 | 6·310 | 6·309 | 24·9 | 6·310 | 0·002 |

certain tasks on the two different systems. To calibrate an electrode using a manual pH meter takes around 4 min of operator time per calibration session, while it takes an operator less than 1 min per day to set up the automated pH system to calibrate automatically every hour. Note that the actual calibration process takes about the same length of time on each system, but no action needs to be taken by the user of the automated pH system, apart from daily changing the buffer solutions. Sample measurement is also faster using the automated pH system, because the operator does not need to devote any time to electrode washing and handling. In this study the operator spent some 17 min per day to measure an average of six samples using the manual pH meter, but only 44 s to make the same measurements using the automated system, as other tasks could be done while

*Table 3. pH measurements of tomato ketchup with a Ag|AgCl sleeve junction electrode.*

| Date | pH 1 | pH 2 | pH 3 | Average temp (°C) | Mean | SD |
|---|---|---|---|---|---|---|
| 13/2/97 | 3·464 | 3·495 | 3·506 | 19·9 | 3·488 | 0·022 |
| 13/2/97 | 3·490 | 3·494 | 3·515 | 21·6 | 3·500 | 0·013 |
| 13/2/97 | 3·482 | 3·502 | 3·512 | 22·9 | 3·499 | 0·015 |
| 13/2/97 | 3·485 | 3·495 | 3·512 | 23·0 | 3·497 | 0·014 |
| 13/2/97 | 3·464 | 3·465 | 3·485 | 23·7 | 3·471 | 0·012 |
| 13/2/97 | 3·506 | 3·500 | 3·502 | 24·1 | 3·503 | 0·003 |
| 13/2/97 | 3·506 | 3·505 | 3·506 | 24·3 | 3·506 | 0·001 |
| 14/2/97 | 3·469 | 3·472 | 3·479 | 20·8 | 3·473 | 0·005 |
| 14/2/97 | 3·479 | 3·477 | 3·483 | 21·2 | 3·480 | 0·003 |
| 14/2/97 | 3·490 | 3·488 | 3·494 | 22·2 | 3·491 | 0·003 |
| 14/2/97 | 3·488 | 3·496 | 3·501 | 22·8 | 3·495 | 0·007 |
| 14/2/97 | 3·493 | 3·501 | 3·504 | 23·3 | 3·499 | 0·006 |
| 14/2/97 | 3·498 | 3·498 | 3·507 | 23·6 | 3·501 | 0·005 |
| 14/2/97 | 3·495 | 3·497 | 3·489 | 23·9 | 3·494 | 0·004 |
| 14/2/97 | 3·492 | 3·502 | 3·502 | 24·0 | 3·499 | 0·006 |
| 14/2/97 | 3·496 | 3·485 | 3·499 | 23·8 | 3·493 | 0·007 |

*Table 4. pH measurements of a water-based automotive paint with a Ag|AgCl sleeve junction electrode.*

| Date | pH 1 | pH 2 | pH 3 | Average temp (°C) | Mean | SD |
|---|---|---|---|---|---|---|
| 14/2/97 | 6·186 | 6·190 | 6·200 | 21·5 | 6·192 | 0·007 |
| 14/2/97 | 6·183 | 6·191 | 6·195 | 22·1 | 6·190 | 0·006 |
| 14/2/97 | 6·132 | 6·137 | 6·140 | 23·9 | 6·136 | 0·004 |

*Table 5. Calibration data from automated pH measurement system.*

| Date | Time | Temp in pH 7 (°C) | mV in pH 7 | Slope %* (pH 4–pH 7) | Slope %* (pH 7–pH 10) |
|---|---|---|---|---|---|
| 18/2/97 | 9.56 a.m. | 23·1 | −8·52 | 98·44 | 97·34 |
| 18/2/97 | 10.56 a.m. | 24·5 | −8·77 | 98·36 | 97·32 |
| 18/2/97 | 11.56 a.m. | 25·6 | −8·92 | 98·36 | 97·33 |
| 19/2/97 | 10.56 a.m. | 23·9 | −9·38 | 98·71 | 97·70 |
| 19/2/97 | 11.56 a.m. | 25·0 | −10·07 | 98·76 | 97·53 |
| 19/2/97 | 12.56 p.m. | 25·8 | −10·81 | 98·80 | 97·41 |
| 20/2/97 | 9.56 a.m. | 23·1 | −8·87 | 98·54 | 97·99 |
| 20/2/97 | 10.56 a.m. | 24·3 | −8·45 | 98·29 | 98·32 |
| 20/2/97 | 11.56 a.m. | 25·5 | −8·65 | 98·35 | 98·19 |
| 21/2/97 | 9.56 a.m. | 24·1 | −8·72 | 98·52 | 98·00 |
| 21/2/97 | 10.56 a.m. | 25·4 | −10·08 | 98·61 | 97·39 |
| 21/2/97 | 12.04 p.m. | 26·4 | −10·46 | 98·78 | 97·38 |

* % of ideal Nernst slope (59·16 mV/pH at 25°C).

the system was running. Manual measurement thus required over 20 times as much operator time as the automated system.

Tables 9–12 show the results for the four samples for the two systems. The samples were measured in two separate sessions, each time in triplicate. The mean and standard deviation was calculated for each sample. The automated pH system gave a lower standard deviation for two of the

*Table 6. Calibration data from manual pH meter.*

| Date | Time | Temp in pH 7 (°C) | mV in pH 7 | Slope %* (pH4–pH10) |
|---|---|---|---|---|
| 18/2/97 | 9.55 a.m. | 18·5 | −8·5 | 98·2 |
| 18/2/97 | 11.10 a.m. | 19·2 | −8·4 | 98·3 |
| 18/2/97 | 11.22 a.m. | 19·1 | −9·1 | 97·9 |
| 19/2/97 | 10.47 a.m. | 19·2 | −10·4 | 97·9 |
| 19/2/97 | 12.00 noon | 19·8 | −10·3 | 97·8 |
| 19/2/97 | 12.15 p.m. | 19·7 | −14·1 | 97·3 |
| 20/2/97 | 10.10 a.m. | 19·2 | −10·6 | 97·9 |
| 20/2/97 | 12.05 p.m. | 19·9 | −11·0 | 97·8 |
| 20/2/97 | 12.20 p.m. | 20·1 | −12·7 | 97·4 |
| 21/2/97 | 10.05 a.m. | 19·3 | −11·3 | 98·0 |
| 21/2/97 | 12.22 p.m. | 20·2 | −10·9 | 97·8 |
| 21/2/97 | 12.40 p.m. | 20·1 | −12·8 | 97·6 |

* % of ideal Nernst slope (59·16 mV/pH at 25°C).

*Table 7. Comparison of volume of buffers used.*

| Date | Manual meter pH 4 | Manual meter pH 7 | Manual meter pH 10 | Automated pH system pH 4 | Automated pH system pH 7 | Automated pH system pH 10 |
|---|---|---|---|---|---|---|
| 18/2/97 | 50 ml | 50 ml | 50 ml | 20 ml | 20 ml | 20 ml |
| 19/2/97 | 100 ml* | 50 ml | 50 ml | 20 ml | 20 ml | 20 ml |
| 20/2/97 | 50 ml | 50 ml | 50 ml | 20 ml | 20 ml | 20 ml |
| 21/2/97 | 50 ml | 50 ml | 50 ml | 20 ml | 20 ml | 20 ml |
| Total | 250 ml | 200 ml | 200 ml | 80 ml | 80 ml | 80 ml |

* Beaker knocked over.

*Table 8. Comparison of time spent by user.*

| | *Manual meter* | | | *Automated pH system* | | |
|---|---|---|---|---|---|---|
| *Date* | *Calibration* | *Buffers* | *Samples* | *Calibration* | *Buffers* | *Samples* |
| 18/2/97 | 5 min 24 s | 1 min 53 s | 2 min 57 s | 0 min | 34 s | 20 s |
| | 4 min 34 s | | 4 min 4 s | | | 20 s |
| | 4 min 48 s | | (6 measurements, mouthwash) | | | (6 measurements mouthwash) |
| 19/2/97 | 4 min 38 s | 1 min | 7 min 50 s | 0 min | 30 s | 20 s |
| | 4 min 28 s | | 8 min 2 s | | | 20 s |
| | 5 min 8 s | | (6 measurements, ketchup) | | | (6 measurements, ketchup) |
| 20/2/97 | 3 min 38 s | 57 s | 11 min 33 s | 0 min | 29 s | 35 s |
| | 3 min 47 s | | 9 min 9 s | | | 20 s |
| | 4 min 29 s | | (6 measurements, shampoo) | | | (6 measurements, shampoo) |
| 21/2/97 | 3 min 41 s | 1 min | 14 min 3 s | 0 min | 33 s | 20 s |
| | 3 min 36 s | | 12 min 8 s | | | 20 s |
| | 3 min 59 s | | (6 measurements, paint) | | | (6 measurements, paint) |
| Total | 52 min 10 s | 4 min 50 s | 69 min 46 s | 0 min | 2 min 6 s | 2 min 55 s |
| Daily average | 13 min 2 s | 1 min 12 s | 17 min 26 s | 0 min | 31 s | 44 s |

samples, mouthwash and shampoo, and produced a similar standard deviation as the manual meter for the ketchup sample. The standard deviation for the paint sample was slightly higher for the automated pH system than for the manual pH meter. This was a difficult sample to measure due to the presence of fine particulate material which settled out during measurement with the manual pH meter.

### Conclusions

The automated pH system is easy to programme with standard methods and has a choice of calibration protocols. This ensures that measurements are always carried out under the same conditions, eliminating human error and bias. Calibration data can be viewed in graph form which makes trends easy to spot, provides a long-term picture of the electrode performance and gives an early warning of electrode failure.

The automated pH system is economical to use as the volume of buffers required is less than half that required in a manual system, and electrodes are less likely to be broken through mishandling. The requirement for labour is also reduced, because the user only needs to spend a minute or so in the morning to change the buffers and wash waters, and the system will calibrate automatically through the day. Once a satisfactory method has been programmed, measuring a sample requires very little effort from the user. The audit trail logs and print-outs will reduce the amount of manual note-keeping that has to be done by the user. Results obtained using the automated system are comparable in precision and accuracy with the manual system, but considerably less operator time is required to use the automated pH system.

*Table 9a. pH measurements of mouthwash with automated pH system.*

| *Date* | *Time* | *pH* | *Temp* (°C) | *pH* | *Temp* (°C) | *pH* | *Temp* (°C) | *Mean* | *SD* |
|---|---|---|---|---|---|---|---|---|---|
| 18/2/97 | 10.19 a.m. | 6·918 | 21·9 | 6·912 | 21·7 | 6·916 | 21·7 | 6·915 | 0·003 |
| 18/2/97 | 11.32 a.m. | 6·912 | 22·4 | 6·908 | 22·3 | 6·911 | 22·3 | 6·910 | 0·002 |

Mean = 6·913, SD = 0·001, $N = 6$.

*Table 9b. pH measurements of mouthwash with manual pH meter.*

| *Date* | *Time* | *pH* | *Temp* (°C) | *pH* | *Temp* (°C) | *pH* | *Temp* (°C) | *Mean* | *SD* |
|---|---|---|---|---|---|---|---|---|---|
| 18/2/97 | 10.00 a.m. | 6·955 | 19·8 | 6·942 | 19·6 | 6·948 | 19·8 | 6·948 | 0·004 |
| 18/2/97 | 11.15 a.m. | 6·952 | 20·6 | 6·950 | 20·4 | 6·959 | 20·2 | 6·954 | 0·005 |

Mean = 6·951, SD = 0·006, $N = 6$.

*Table 10a. pH measurements of tomato ketchup with automated pH system.*

| *Date* | *Time* | *pH* | *Temp* (°C) | *pH* | *Temp* (°C) | *pH* | *Temp* (°C) | *Mean* | *SD* |
|---|---|---|---|---|---|---|---|---|---|
| 19/2/97 | 11.20 a.m. | 3·479 | 21·9 | 3·482 | 21·8 | 3·490 | 21·8 | 3·484 | 0·006 |
| 19/2/97 | 12.40 p.m. | 3·469 | 22·6 | 3·474 | 22·6 | 3·477 | 22·5 | 3·473 | 0·004 |

Mean = 3·479, SD = 0·007, $N = 6$.

*Table 10b. pH measurements of tomato ketchup with manual pH meter.*

| *Date* | *Time* | *pH* | *Temp* (°C) | *pH* | *Temp* (°C) | *pH* | *Temp* (°C) | *Mean* | *SD* |
|---|---|---|---|---|---|---|---|---|---|
| 19/2/97 | 10.55 a.m. | 3·495 | 19·3 | 3·493 | 19·3 | 3·492 | 19·5 | 3·493 | 0·002 |
| 19/2/97 | 12.07 p.m. | 3·505 | 21·0 | 3·493 | 20·8 | 3·482 | 20·5 | 3·493 | 0·012 |

Mean = 3·493, SD = 0·007, $N = 6$.

*Table 11a. pH measurements of shampoo with automated pH system*

| *Date* | *Time* | *pH* | *Temp* (°C) | *pH* | *Temp* (°C) | *pH* | *Temp* (°C) | *Mean* | *SD* |
|---|---|---|---|---|---|---|---|---|---|
| 20/2/97 | 10.45 a.m. | 6·171 | 21·6 | 6·156 | 21·3 | 6·164 | 21·3 | 6·164 | 0·008 |
| 20/2/97 | 11.45 p.m. | 6·168 | 22·6 | 6·168 | 21·9 | 6·165 | 22·2 | 6·167 | 0·002 |

Mean = 6·165, SD = 0·005, $N = 6$.

*Table 11b. pH measurements of shampoo with manual pH meter.*

| *Date* | *Time* | *pH* | *Temp* (°C) | *pH* | *Temp* (°C) | *pH* | *Temp* (°C) | *Mean* | *SD* |
|---|---|---|---|---|---|---|---|---|---|
| 20/2/97 | 10.15 a.m. | 6·239 | 18·8 | 6·212 | 19·0 | 6·195 | 19·0 | 6·215 | 0·022 |
| 20/2/97 | 12.10 p.m. | 6·227 | 20·9 | 6·202 | 20·2 | 6·201 | 20·3 | 6·210 | 0·015 |

Mean = 6·213, SD = 0·017, $N = 6$.

*Table 12a. pH measurements of water-based automotive paint with automated pH system*

| *Date* | *Time* | *pH* | *Temp* (°C) | *pH* | *Temp* (°C) | *pH* | *Temp* (°C) | *Mean* | *SD* |
|---|---|---|---|---|---|---|---|---|---|
| 21/2/97 | 10.33 a.m. | 6·151 | 22·2 | 6·166 | 22·0 | 6·170 | 22·0 | 6·162 | 0·010 |
| 21/2/97 | 11.55 a.m. | 6·118 | 23·4 | 6·133 | 23·2 | 6·139 | 23·3 | 6·130 | 0·011 |

Mean = 6·146, SD = 0·020, $N = 6$.

*Table 12b. pH measurement of water-based automotive paint with manual pH meter.*

| *Date* | *Time* | *pH* | *Temp* (°C) | *pH* | *Temp* (°C) | *pH* | *Temp* (°C) | *Mean* | *SD* |
|---|---|---|---|---|---|---|---|---|---|
| 21/2/97 | 10.15 a.m. | 6·171 | 21·1 | 6·174 | 21·5 | 6·185 | 21·1 | 6·177 | 0·007 |
| 21/2/97 | 12.27 p.m. | 6·143 | 22·0 | 6·149 | 21·8 | 6·168 | 21·8 | 6·153 | 0·013 |

Mean = 6·165, SD = 0·016, $N = 6$.

### Acknowledgements

The authors would like to thank Mark Taylor for his help measuring the samples with the manual pH meters and Richard Bhella for writing some of the sample methods.

### References

1. Bates, R. G., *Determination of pH—Theory and Practice* (John Wiley,New York, 1973).
2. Clark Westcott, C., *pH Measurements* (Academic Press, San Diego, 1978).
3. Galster, H., *pH Measurement: Fundamentals, Methods, Applications, Instrumentation* (VCH, Weinheim, 1991).
4. Gardner, M. J., Ravenscroft, J. E. and Ackers, C., *Talanta*, **44** (1997), 111–117.
5. British Standard, BS 6068, Section 2.50, ISO 10523 (1994).
6. Frant, M. S., *American Laboratory*, **27** (1995), 11.
7. Torres, P., García-Mesa, J. A., Luque de Castro, M. D. and Valcárel, M., *Fresenius J. Anal. Chem.*, **346** (1993), 704–706.
8. Brenes, N., Quigley, M. N. and Reid,W. S., *Analytica Chimica Acta.*, **310** (1995), 319–327.
9. Covington, A. K., Bütikofer, H. P., Camoes, M. F. G. F. C., Ferra, M. I. A. and Rebelo, M. J. F., *Pure & Appl. Chem.*, **57** (1985), 893.

